# Supplementary figures and images for: A handheld luminometer with sub-attomole limit of detection for distributed applications in global health
Source: PLOS Glob Public Health. 2024 Feb 21;4(2):e0002766. doi: 10.1371/journal.pgph.0002766 (PMC10881016; doi:10.1371/journal.pgph.0002766)

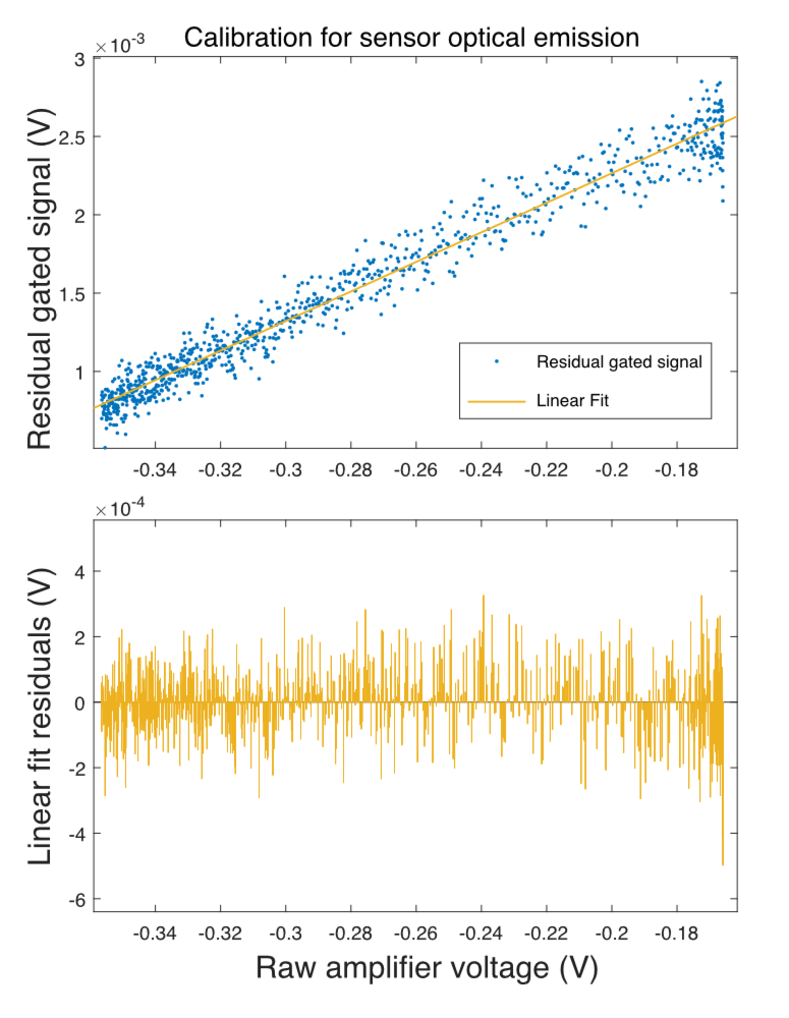

Supplement: S1 Fig — Top: Scatter plot of residual gated signal, collected during an ambient temperature ramp from 40°C to 4°C, under dark conditions. The residual signal due to the ECC effect is observed to be directly proportional to the raw amplifier voltage, which is in turn linearly proportional to sensor’s dark current. The shutter flag modulates the ECC coupling ratio from the sensor back to itself, by blocking and unblocking the reflective sample cavity. The magnitude of the effect represents approximately 150 RLU of correlated error over the recorded temperature range. Bottom: Residuals from the linear fit to the data, showing no evidence of bias across the range of sampled temperatures. (TIF) [file pgph.0002766.s001.tif]

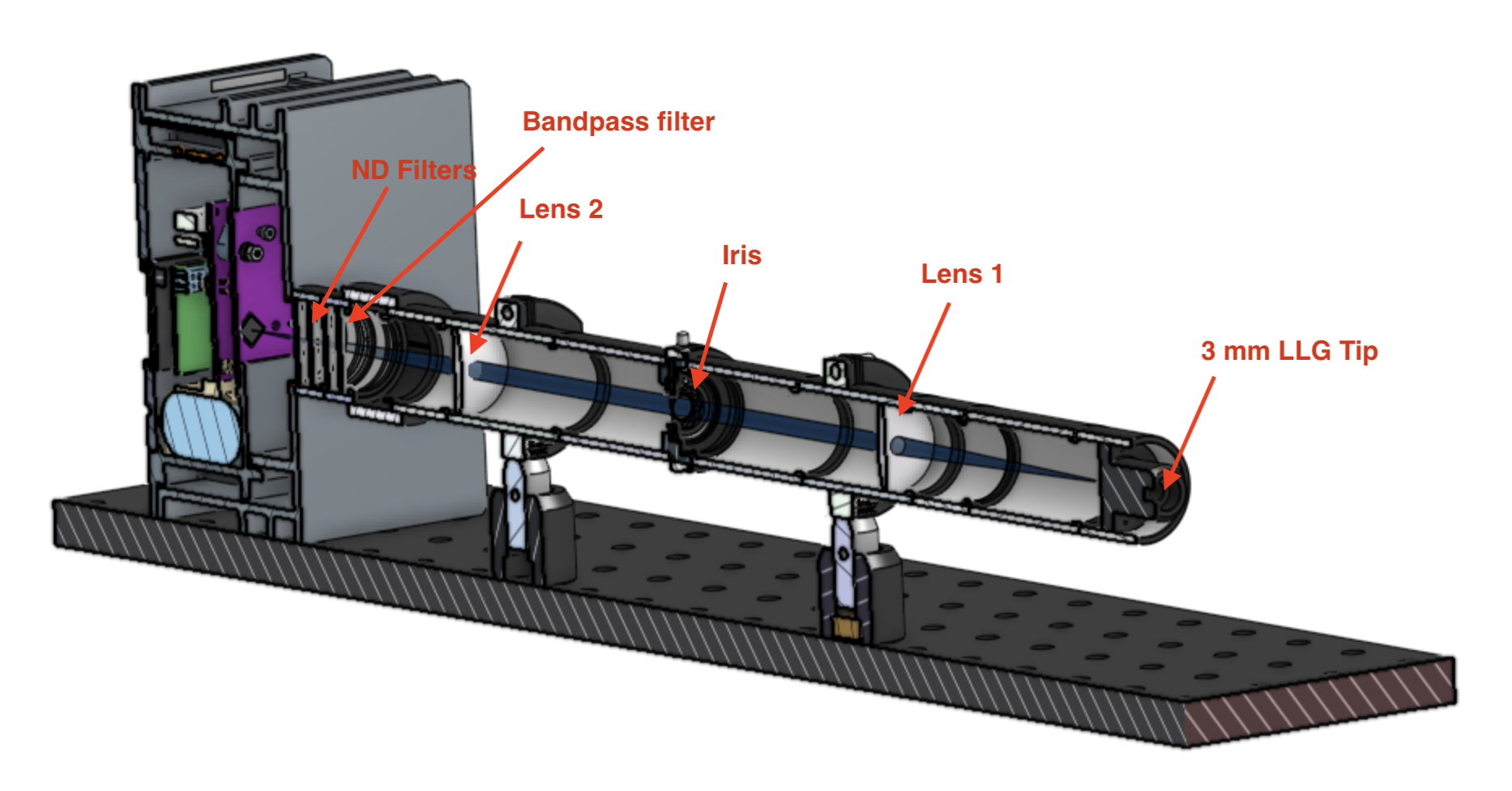

Supplement: S2 Fig — A 3D CAD model of the radiometric test setup is shown as a cross-section view. The optical path consisted of a 4f relay, imaging the tip of a 3 mm Liquid Light Guide (LLG) onto the luminometer sensor at 1× magnification. Variable numbers of neutral density filters were added or removed as required to vary the optical power, along with the lamp’s percentage output setting (0–100%). The lens relay was constructed using two 100 mm focusing lenses (Thorlabs LA1509-A), and an adjustable iris (fixed at 2 mm) to restrict the NA of the optical path. A 25 nm FWHM spectral bandpass filter (Semrock FF02-475/50-25) was employed to approximately match the wavelength of the lamp’s spectrum to that of nanoluciferase emission (460 nm). The incident optical power at the luminometer sensor was varied between 0.1 fW to nearly 1E5 fW. (TIF) [file pgph.0002766.s002.tif]

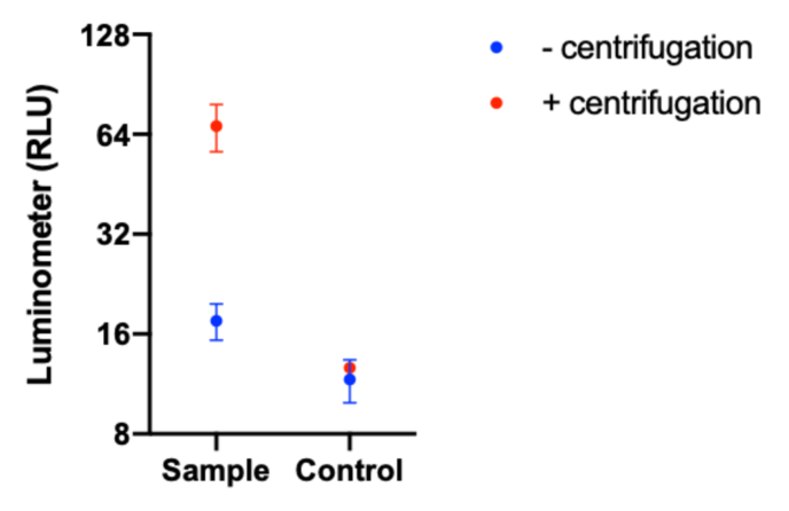

Supplement: S3 Fig — The spLUC assay was run as previously described and the RLU signal was compared with and without centrifugation of the red cells. Centrifugation increased the signal by 5-fold for this sample from a vaccinated volunteer. (TIF) [file pgph.0002766.s003.tif]

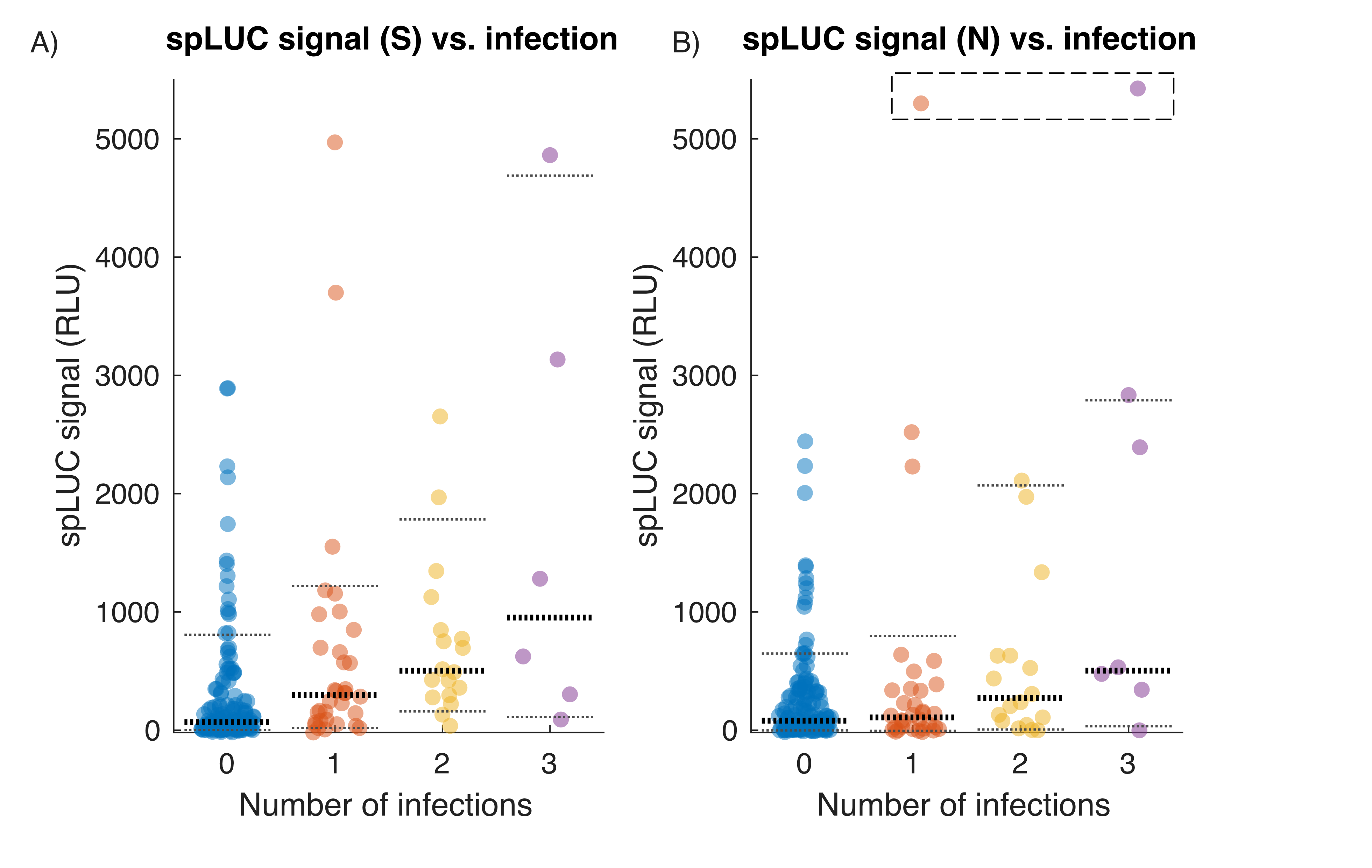

Supplement: S4 Fig — A) S signal vs. self-reported number of infections. B) N signal vs. self-reported number of infections. In both plots, bold dotted lines denote the median of each distribution, and fine dotted lines denote the 10th and 90th percentiles of each distribution. In B), two outlier data points (the same points from Fig 5C in the main text) were artificially lowered into the display range (dashed box), which was limited to 5500 for clarity. (TIF) [file pgph.0002766.s004.tif]

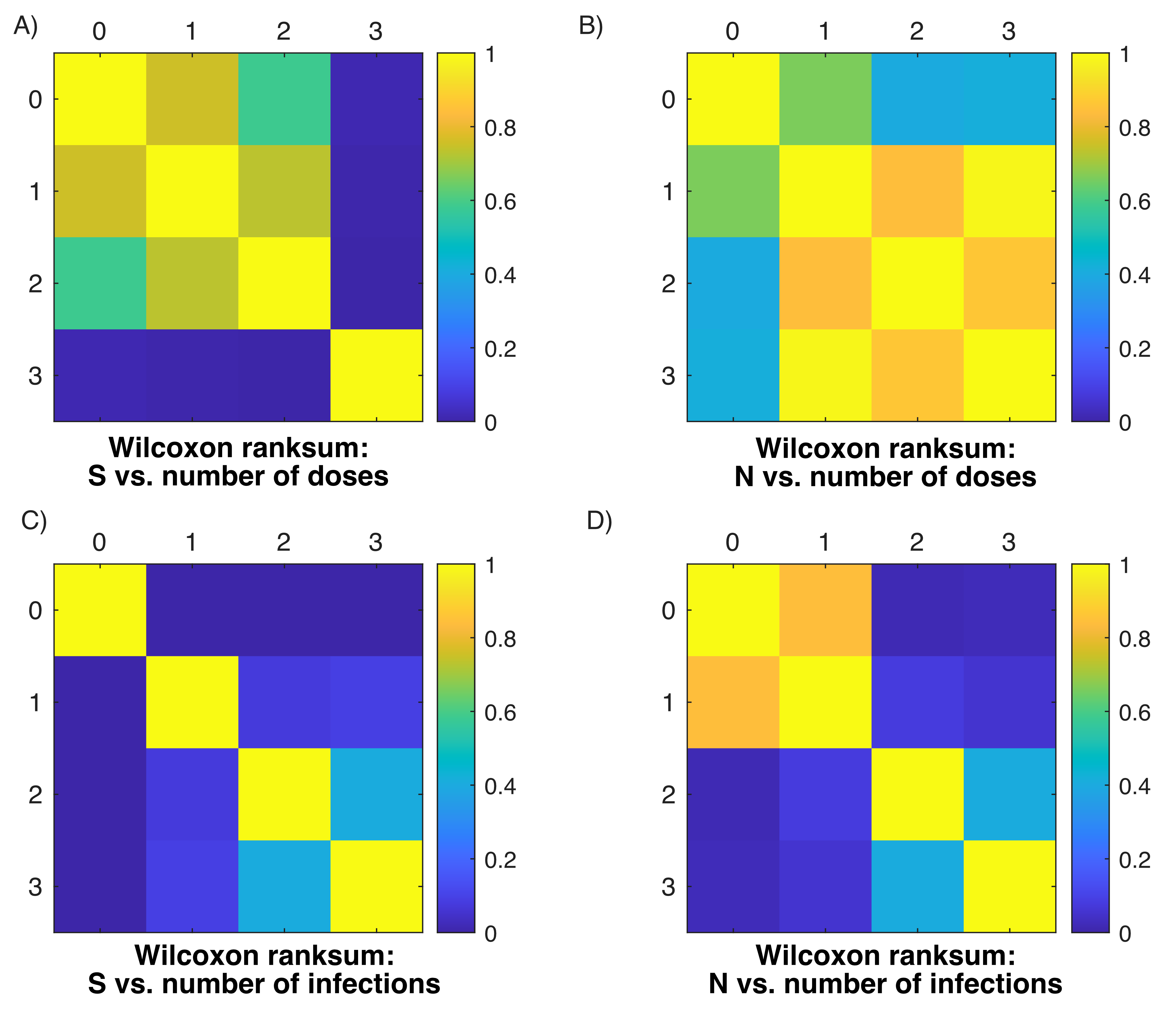

Supplement: S5 Fig — Ranksum coefficients denote the P-values of a two-sided Wilcoxon ranksum test for the median similarity of the sampled distributions, where a value of 1.0 indicates an identical distribution median. Diagonal entries in each matrix compare the same data to itself and all have a value of 1.0. Off-diagonal entries compare distributions from sets of participants with different reported numbers of either doses (A and B) or infections (C and D). Coefficients for the S sensor are shown in A) and C), and for the N sensor in B) and D). (TIF) [file pgph.0002766.s005.tif]

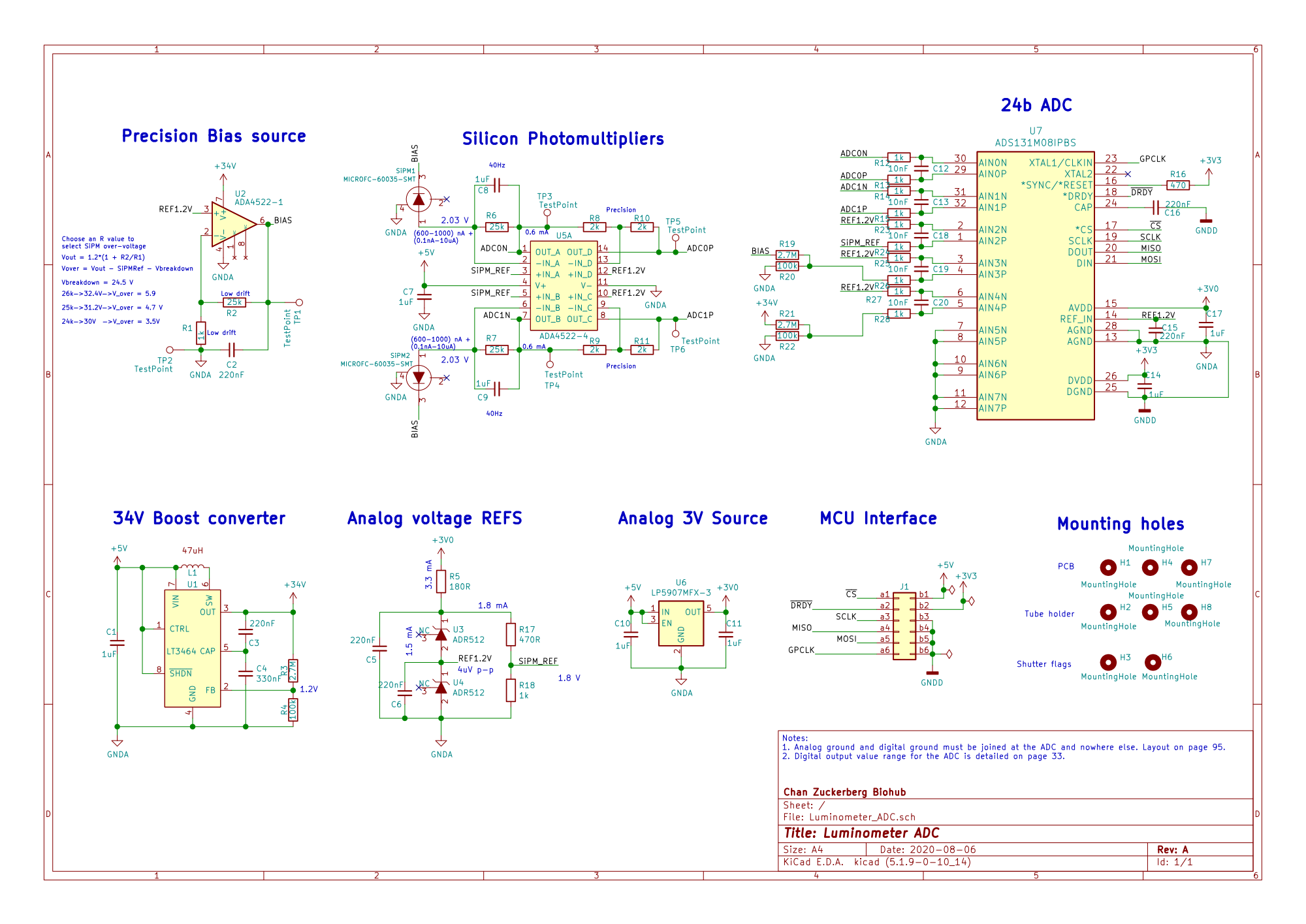

Supplement: S6 Fig — Clockwise, from upper left: the precision bias source uses a zero-drift amplifier and precision low-drift resistors to set the SiPM bias voltage, which controls gain and photon detection efficiency of the sensors. This bias source is powered by an on-board boost converter to generate a 34V supply. The SiPM sensors themselves are read out by fully-differential, zero-drift transimpedance amplifiers, and routed with short traces to a 24-bit differential-input ADC with “global chop” DC bias elimination and digital over-sampling for anti-aliasing. The transimpedance amplifier gain was chosen to position the maximum expected signals near the point of saturation of the amplifier, with parallel capacitors low-pass filtering the bandwidth of the amplified signal. Precision voltage reference sources were used for both the ADC and the differential amplifier. The ADC digitized the signals using an SPI protocol routed directly to a board-to-board interconnect. (TIF) [file pgph.0002766.s006.tif]

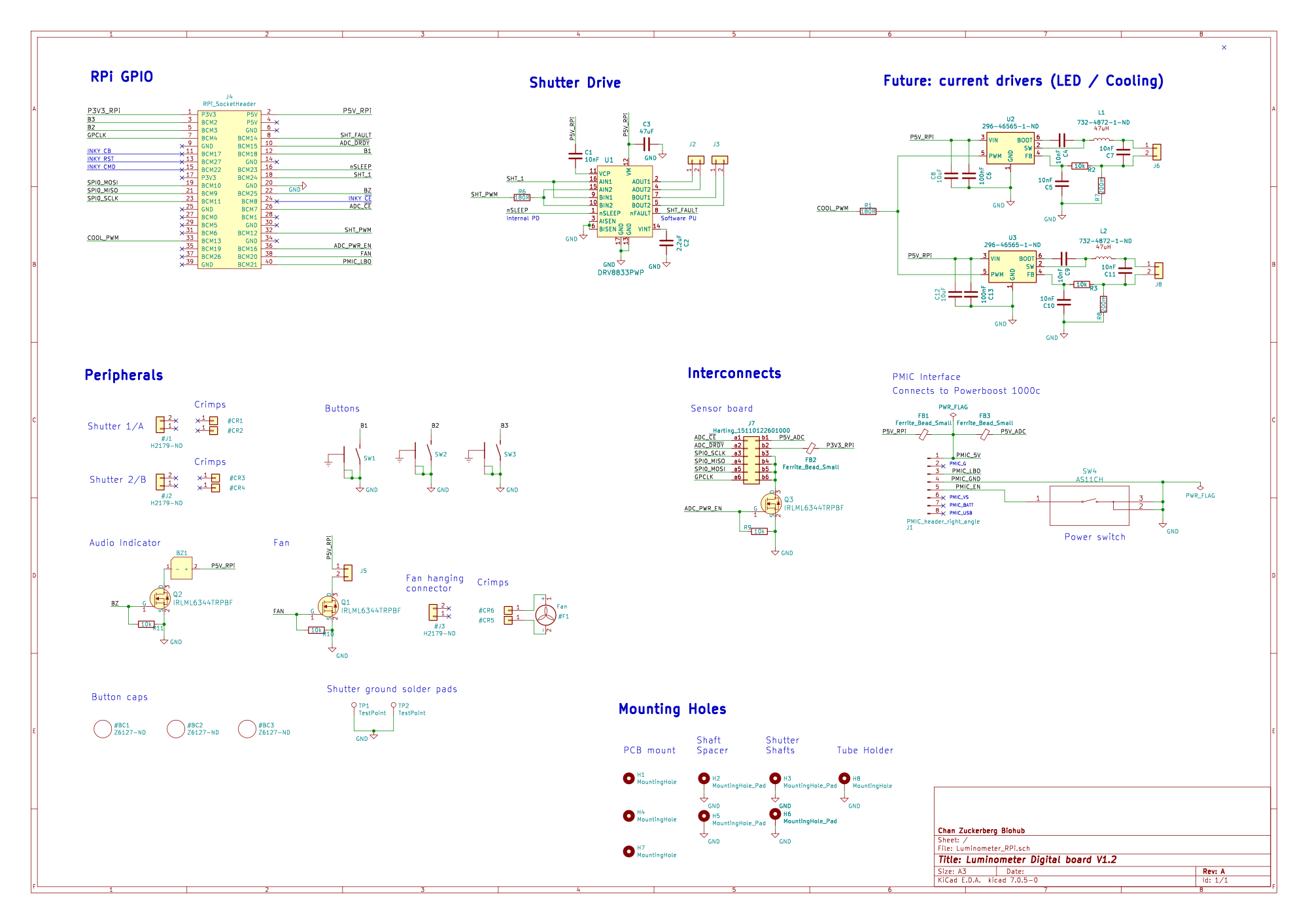

Supplement: S7 Fig — Clockwise, from upper left: Connection to the Raspberry Pi zero GPIO header, with labeled pins. H-Bridge circuit for driving the mechanical shutter system. Unused: Buck converter LED driver chips for potential future usage in fluorescence applications, or sensor temperature stabilization (Peltier). Mechanical interconnects: Board to board interconnect (J7) connecting to the analog sensor board, and jumper cables (J1) to the power management board, and the power switch (SW4). Peripherals include: shutter jumper cables (#J1, #J2), user interface buttons (SW1-3), audio indicator (BZ1), fan connector (J5) and its cable (#J3), button caps (#BC1-2) and ground electrodes for the shutter system (TP1-2). Mounting holes were used for PCB mechanical mounting. (TIF) [file pgph.0002766.s007.tif]

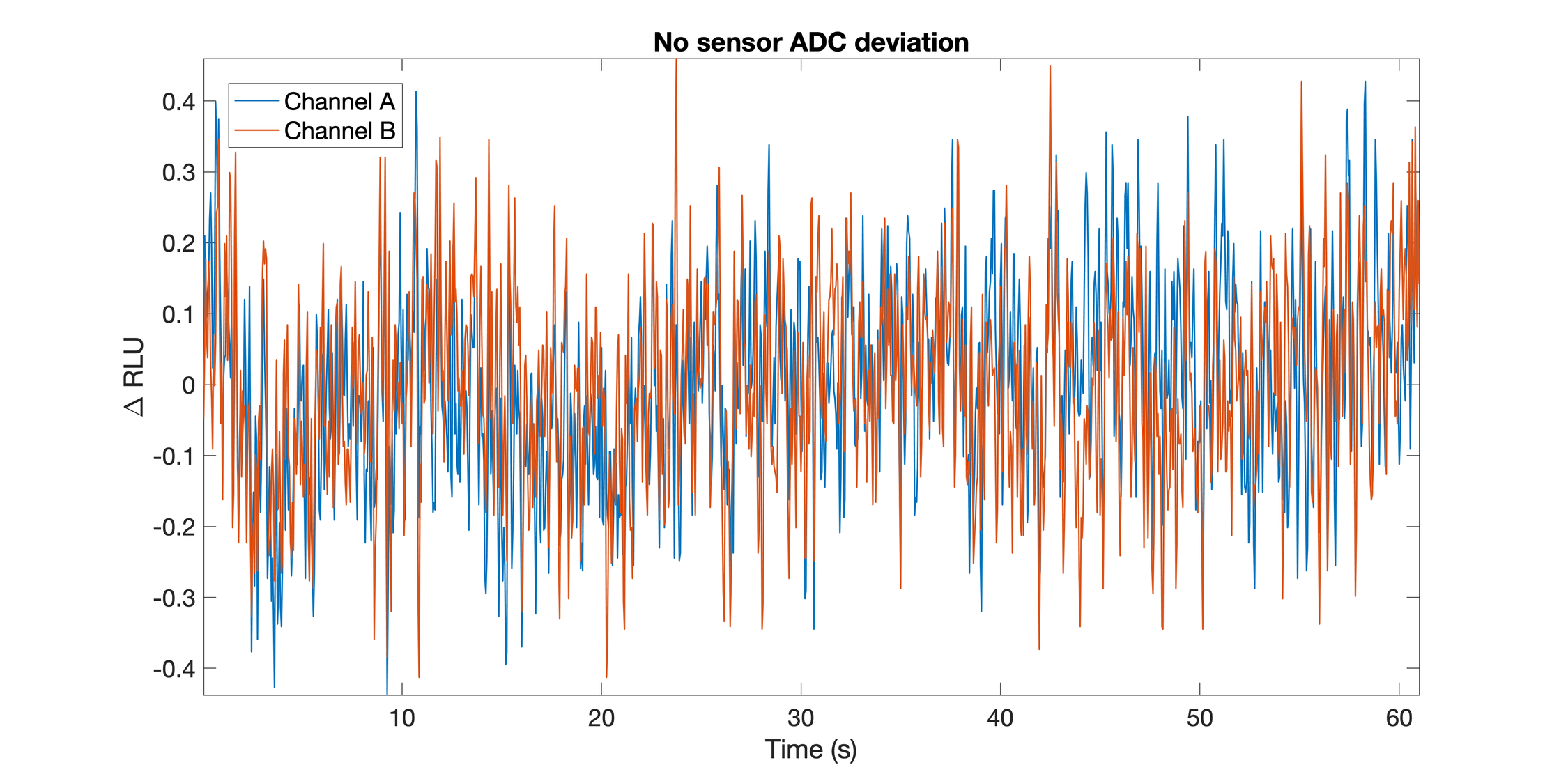

Supplement: S8 Fig — Measurements of the transimpedance amplifier noise were acquired prior to installation of the SiPM sensors, in order to characterize the baseline noise in the absence of sensor current. The resulting traces exhibited a total standard deviation on the order of 0.1 RLU at a sampling rate of 20 Hz. With temporal averaging over a period of 30–60 seconds, this noise is further reduced and is more than 100-fold lower than any expected luminescence signals (> 1 RLU). The ADC discretization limit was 7.2 nV, or 0.003 RLU. (TIF) [file pgph.0002766.s008.tif]
