# Supplementary material for: A handheld luminometer with sub-attomole limit of detection for distributed applications in global health
Source: PLOS Glob Public Health. 2024 Feb 21;4(2):e0002766. doi: 10.1371/journal.pgph.0002766 (PMC10881016; doi:10.1371/journal.pgph.0002766)
Supplement: S1 Table — (DOCX) [file pgph.0002766.s009.docx]

# S1 Table: Commercial and academic luminometer Limits of Detection (LODs)

LODs are quoted either in terms of moles of luciferase, moles of ATP, radiometric power, or other units, with all conversions dependent on several assumptions. Conversion between the two luciferase-based systems of reporting depends on the enzyme used (nanoluciferase vs firefly luciferase) and the assay’s kinetic conditions. Assuming flash-mode kinetics, the catalytic rate *k_cat_* of firefly luciferase was previously reported to be approximately 1.6 s^-1^ (1). Note also that conversion between enzymatic standards and radiometric power depends on both assay conditions as well as collection efficiency and detector size. Smaller detectors can exhibit superior radiometric LODs due to their lower dark current, while light collection efficiency and LOD for enzymatic signals will be reduced, as detailed in Supplementary Note 2. We estimate that 1E-17 moles of ATP is approximately equivalent to 1.2E-19 moles of firefly luciferase, with each assay operating in flash mode kinetics and the other component in saturation, and with a signal half-life of 30 seconds, although differing assumptions will produce differing results.

All the above factors must be considered when comparing across methods of reporting, none of which capture every aspect relevant to all applications.

| **Make/ref #** | **Model/Author** | **Sensor** | **Portable** | **Battery** | **Limit of detection** | **Cost** |
| --- | --- | --- | --- | --- | --- | --- |
| Promega | GloMax | PMT | No | No | 3 E-21 moles of luciferase | $16,313 |
| Berthold | Junior | PMT | Semi^a^ | Yes | 5 E-17 moles of ATP | $8,000 |
| BMG | Omega | PMT | No | No | 2 E-17 moles of ATP | $13,560 |
| Tecan | Infinite M-Plex | PMT | No | No | 1.2 E-17 moles of ATP | $36,000 |
| 3M | LX25 | Not reported | Yes | Yes | 5 E-16 moles of ATP | $3,409 |
| (2) | Bunce et al. | Film | No | No | 5E-13 moles of luminol | Not reported |
| (3) | Porakishvili et al. | Cooled CCD | No | No | Conversion not known | £3,000 |
| (4) | Bunce et al. | PIN Photodiode | Yes | Yes | 5E-13 moles of ATP | Not reported |
| (5) | Roda et al. | Smartphone | Yes | Yes | 1E-4 moles/L lactate | Cost of phone |
| (6) | Kim et al. | Smartphone | Yes | Yes | 1-10 pW | Cost of phone |
| (7) | Li et al | SiPM  (1 mm^2^) | Not reported | No | 0.6 fW ^b,c,d^ | Not reported |
| (8) | Jung et al. | SiPM  (9 mm^2^) | Yes | Yes | 100 fW^b,c^ | Not reported |
| (9) | Calabretta et al. | SiPM  (1.7 mm^2^) | Yes | No | 9E-15 moles of luciferase^b^ | Not reported |
| (10) | Baszczyk et al. | SiPM  (1 mm^2^) | Yes | No | 73 fW ^b,d^ | Not reported |
| **This work** | **CZ Biohub SF** | **SiPM**  **(36 mm^2^)** | **Yes^e^** | **Yes** | **1.6E-19 moles of luciferase or**  **1 fW** | **< $1,000** |

^a^ Mass is 2 kg and a suitcase-sized carrying case is required for transport.

^b^ Sensor active area is small, limiting applications in luminescence detection.

^c^ Active cooling used

^d^ Computed from quoted limit of detection in counts per second, using a wavelength of 460 nm.

^e^ Mass is 515 g, and outer dimensions are 108 x 122 x 80 mm (w x h x d)

# References

1. Branchini BR, Magyar RA, Murtiashaw MH, Anderson SM, Zimmer M. Site-Directed Mutagenesis of Histidine 245 in Firefly Luciferase:  A Proposed Model of the Active Site. Biochemistry. 1998 Nov 1;37(44):15311–9.

2. Bunce RA, Thorpe GHG, Gibbons JEC, Killeen PR, Ogden G, Kricka LJ, et al. Camera luminometer for use with luminescent assays. Analyst. 1985 Jan 1;110(6):657–63.

3. Porakishvili N, Fordham JLA, Charrel M, Delves PJ, Lund T, Roitt IM. A low budget luminometer for sensitive chemiluminescent immunoassays. 2000;8.

4. Marks K, Killeen P, Goundry J, Gibbons J, Bunce R. A portable silicon photodiode luminometer. J Biolumin Chemilumin. 1987;1(3):173–9.

5. Roda A, Guardigli M, Calabria D, Calabretta MM, Cevenini L, Michelini E. A 3D-printed device for a smartphone-based chemiluminescence biosensor for lactate in oral fluid and sweat. The Analyst. 2014 Sep 26;139(24):6494–501.

6. Kim H, Jung Y, Doh IJ, Lozano-Mahecha RA, Applegate B, Bae E. Smartphone-based low light detection for bioluminescence application. Sci Rep. 2017 Jan 9;7(1):40203.

7. Li H, Lopes N, Moser S, Sayler G, Ripp S. Silicon photomultiplier (SPM) detection of low-level bioluminescence for the development of deployable whole-cell biosensors: Possibilities and limitations. Biosens Bioelectron. 2012 Mar 15;33(1):299–303.

8. Jung Y, Coronel-Aguilera C, Doh IJ, Min HJ, Lim T, Applegate BM, et al. Design and application of a portable luminometer for bioluminescence detection. Appl Opt. 2020 Jan 20;59(3):801–10.

9. Calabretta MM, Montali L, Lopreside A, Fragapane F, Iacoangeli F, Roda A, et al. Ultrasensitive On-Field Luminescence Detection Using a Low-Cost Silicon Photomultiplier Device. Anal Chem. 2021 May 25;93(20):7388–93.

10. Baszczyk M, Dorosz P, Mik L, Kucewicz W, Reczynski W, Sapor M. A readout circuit dedicated for the detection of chemiluminescence using a silicon photomultiplier. J Instrum. 2018 May;13(05):P05010–P05010.
