# Supplementary material for: A handheld luminometer with sub-attomole limit of detection for distributed applications in global health
Source: PLOS Glob Public Health. 2024 Feb 21;4(2):e0002766. doi: 10.1371/journal.pgph.0002766 (PMC10881016; doi:10.1371/journal.pgph.0002766)
Supplement: S4 File — (DOCX) [file pgph.0002766.s020.docx]

## Supplementary Note 1: *Etendue* and optical collection efficiency

The principle of *Etendue* states that the product of surface area and solid angle of a projected emitting surface does not decrease via transmission through any series of optical elements (1):

$dU=n^{2}dA cos(\theta) d\Omega$

Where *dU* is the differential unit of *Etendue*, *n* is the refractive index of the immersed medium, *dA* is the differential unit of surface area, $\theta$is the projected angle, and $d\Omega$ is the differential unit of solid angle. The implications place practical limits on how much light can be collected by an optical sensor with finite surface area and collection angle, given the source’s optical emission characteristics. If the source is larger than the sensor, then a de-magnified image could be created at the sensor to match its surface area. However, the conservation of Etendue states that to reduce the area of the image, the acceptance angle at the sensor must be proportionally larger than the collection angle at the source.

Equivalently, the radiance of the original emission source (photon flux per unit volume per unit solid angle) cannot be increased by the use of lenses and/or mirrors. Collection optics therefore cannot play a beneficial role in focusing luminescence emission onto a small detector, beyond the result of positioning the sensor immediately adjacent to the sample. However, a reflecting surface can be used to redirect light emitted in the opposite direction from the sensor, which would otherwise be lost.

By contrast, in fluorescence microscopy, since dye molecules generate photons from a nanoscopic volume, optics with large magnification factors can be used to collect and focus emission onto microscopic detectors with extremely low dark current -- for example, a small cluster of cooled EMCCD or sCMOS camera pixels. Since the dark current of such cooled scientific camera pixels is typically less than one electron per pixel per second (2), the detector noise is insignificant in comparison to photon shot noise from the signal itself. Since the emission volume of a typical luminescence sample is tens to hundreds of μL (1μL = 1 mm^3^) in volume, similar use of magnification would require impractically large detectors with proportionally large dark currents. The challenges of luminescence detection are therefore distinct from other low-light applications such as single molecule fluorescence microscopy.

### Optimal sensor size

The optimal sensor size is influenced by the Lambertian emission profile of the sample, the solid angle subtended by the sensor with respect to the sample, and the dark current’s dependence on sensor area. Under the assumption that dark current is proportional to area, we extend equation 1 from the main text to include an integrated Lambertian collection profile (uniform emission per unit solid angle, projected onto the plane of the sensor) of the form $(1-cos(\theta))$, where θ is the half-angle subtended by the sensor. This equation approximates SNR (under the assumption that dark current is much larger than signal current), up to a proportionality constant:

$SNR \sim\frac{1-cos(\theta)}{\sqrt{2y^{2}}}$

Where y is the half-width of the sensor, and r_o_ is the distance between the sample and the sensor. Here, we simplify the calculation by temporarily considering the emitter to be a point source (a proper integral over the sample will have the effect of broadening the peak). By substituting $cos(\theta)=r/h$, where $h=\sqrt{(y^{2}+r^{2})}$ is the hypotenuse of the solid angle, we get:

$SNR \sim\frac{1}{y}-\frac{r}{y\sqrt{{(y}^{2}+r^{2})}}$

Substituting $\eta=\frac{y}{r}$, we obtain the unitless representation proportional to the SNR:

$SNR\sim\left( \frac{1}{\eta}-\frac{1}{\eta\sqrt{\eta^{2}+1}} \right)$

The derivative of the function with respect to y can be set to zero to solve for the optimal value of y, or solved graphically. A result emerges, suggesting that optimal SNR is achieved by using a sensor marginally larger than the sample size. Solved graphically, the optimal sensor-to-sample size ratio is approximately 1.2.


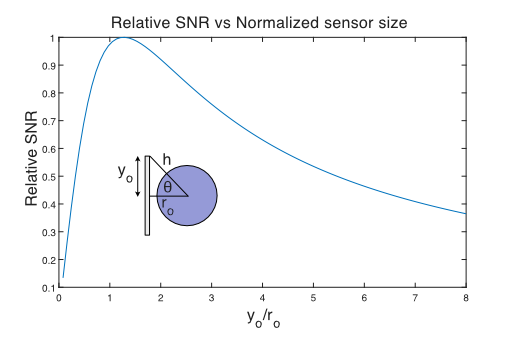


## References

1. Chaves J. Introduction to Nonimaging Optics. CRC Press; 2017. 772 p.

2. andor-zyla-for-physical-sciences-specifications.pdf [Internet]. [cited 2021 Nov 2]. Available from: https://andor.oxinst.com/assets/uploads/products/andor/documents/andor-zyla-for-physical-sciences-specifications.pdf
