# Supplementary material for: A handheld luminometer with sub-attomole limit of detection for distributed applications in global health
Source: PLOS Glob Public Health. 2024 Feb 21;4(2):e0002766. doi: 10.1371/journal.pgph.0002766 (PMC10881016; doi:10.1371/journal.pgph.0002766)
Supplement: Attachment — Submitted filename: Change log.docx [file pgph.0002766.s024.docx]

# Log of changes since medRxiv preprint submission

**Preprint posted May 26, 2023:** <https://www.medrxiv.org/content/10.1101/2023.05.18.23290120v1>

**Changes related to PLOS submission guidelines:**

1. Changed reference style to ‘Vancouver’.
2. Changed line spacing to ‘double-spaced’.
3. Added a cover letter.
4. Created title page with author affiliations as described in submission guidelines.
5. Re-named figures and their references to “Fig 1”, “Fig 2”, etc.
6. Changed location of figure captions to paragraph following first reference.
7. Removed figures from the manuscript file.
8. Exported figures to individual .tiff files and renamed to conform with PLOS conventions.
9. Re-arrangements of Supplementary material:
   a. Re-ordered Supplementary figures to correspond with reference order.
   b. Renamed all Supplementary materials to “S1 Fig”, “S2 Fig”, etc
   c. Removed the original “Supplementary Information” file as all its content is now contained in captions and individual files.
   d. Created S1 Note, merging previous notes 1 and 2.
   e. Converted Supplementary Table 1 to individual file: “S1 Table.docx”.
   f. Converted Supplementary Table 2 to individual file: “S4 Data.xlsx”
   g. Moved Supplementary material captions to end of the manuscript file, under the “Supporting Information” heading.
   h. Convert supplementary data files to .xlsx instead of .pdf file format.

**Other changes:**

- Removed previous Supplementary Figure 5 as it did not add useful information.
- Added clarifying information to the new S2 Data.xlsx file to clarify that device #3 was not functional during that experiment.
- Minor adjustment to alignment of panels b and c in Fig 4 and removed a spurious tiny line segment.
- A small number of very minor grammatical corrections were made to the manuscript text.
- Clarified the per-unit cost of the Analog and Digital PCB assemblies in S1 BOM.xlsx line items (“Total per luminometer” did not change, only the “Pack price” column).
- Corrected misformatted equations after conversion from Google Docs to microsoft word
- Updated author affiliation for R.P.
