# Supplementary material for: A handheld luminometer with sub-attomole limit of detection for distributed applications in global health
Source: PLOS Glob Public Health. 2024 Feb 21;4(2):e0002766. doi: 10.1371/journal.pgph.0002766 (PMC10881016; doi:10.1371/journal.pgph.0002766)
Supplement: Attachment — Submitted filename: Luminometer rebuttal letter plos.docx [file pgph.0002766.s025.docx]

October 27^th^, 2023

PLOS Global Public Health

1265 Battery Street, Suite 200

San Francisco, CA 94111

Dear PLOS Editorial Team and peer reviewers,

We thank you for the time you invested, and for the positive response to our manuscript titled “A handheld luminometer with sub-attomole limit of detection for distributed applications in global health”. We have addressed the points raised by the journal editor as well as by the peer review. Please see our itemized responses to the (italicized) points below:

We noticed that you used “data not shown" in the manuscript. We do not allow these references, as the PLOS data access policy requires that all data be either published with the manuscript or made available in a publicly accessible database. Please amend the supplementary material to include the referenced data or remove the references.

We have removed the statement regarding the extended high temperature stress test of the devices due to the absence of data to present. Although the test itself was valuable, removing the statement does not take away from the manuscript because the downstream applications we demonstrate build on these results and themselves demonstrate the robustness of the design. We thank the editor for helping improve the manuscript in this way.

Reviewer #1: This is a thorough and well written research paper with rigorous validation data in calibration and real sample testing with sound technical contents. In addition, data supported are truly ‘open source’ so that anyone can try to replicate the experiment with relatively low cost.

We are very thankful to Reviewer #1 for their positive comments on our work, and for the time they invested in contributing to the peer review process.

Reviewer #2: The manuscript details a substantial body of work, both in the design of the apparatus as well as the analysis of it's performance and potential short-comings. The reviewer has the following comments, which would perhaps enhance the manuscript.

We thank Reviewer #2 for their overall positive outlook on the manuscript and for their constructive points, adding value to the work. We hope they will find our responses and corresponding changes satisfactory.

1) Limit of detection: Why is the LOD stated for firefly luciferase but not for ATP? A lot of commercial devices in this arena state their LOD for ATP. Whilst it is true that NanoLuc does not need ATP and perhaps there is value in giving information on the LOD with respect to the amount of firefly luciferase, in order to compare devices better, it would be good to get the limit of detection with respect to ATP, at least where firefly luciferase is used.

The reviewer raises a valid point as the units of measurement (and the assay conditions) affect how the reported data can be interpreted and under which circumstances it is valid. Many luminometer manufacturers do advertise their LOD in moles of ATP. Similarly, others report in units of enzyme concentration – for example, Promega – the manufacturer of both the luciferase reagents as well as the most sensitive benchtop luminometers we could find in our online searches. Other units used in academic publications include concentration of luminol, lactate, or radiometric optical power. In all cases, the measurements always depend on other variables, reported to varying degrees: for example, the composition of the assay buffer, the concentration of enzyme (in the ATP-limited case), or the concentration of ATP used (in the enzyme-limited case). We note that authors claiming sensitivities in units of radiometric power must also consider optical collection efficiency as a confounding factor because a laser beam (or other paraxial light source) results in near perfect collection efficiency that is not achieved when measuring luminescent samples with Lambertian emission profiles.

Of all the luminescence based LOD reporting methods, we emphasize that the fundamental distinction resides in the kinetics of the assay, i.e., how quickly the photons are generated per enzyme per unit time and their emission half-life, regardless of whether the assay is enzyme-limited or ATP-limited in design. To span a broad range of scenarios, we chose to report our LOD in three different ways: flash-mode kinetics (Luciferase Assay System with firefly luciferase), glow-mode kinetics (Bright-Glo system with firefly luciferase), and independently, by radiometric power. In doing so, our results cover both commonly encountered assay kinetics regimes, as well as radiometric reporting to explore the fundamental physical properties of the detection system.

The reviewer suggests adding a fourth method of reporting our LOD by instead limiting the quantity of ATP. While appealing to convenience for readers who wish to compare more directly with a subset of device manufacturers, it is important to note that even this method carries its own specifics such as which enzyme and assay buffer were selected, the exposure time used, and how rapidly the solution was mixed prior to measurement. While recognizing the importance of the reviewer’s comment in pointing out the variety of units used in the field, we respectfully assert that our three reporting methods already span a representative range of assay parameters, and exceeds the rigor and standards typically encountered in both commercial and academic device specifications.

2) The dried split-luciferase and other sensors are all stored at -20C. I'm unsure how feasible this makes usage in rural areas. Perhaps information on the life-span of these reagents at 4C and/or room temperature would help in decision making/planning beforehand, for those who wish to use this luminometer in a similar fashion.

We thank the reviewer for raising this important point, as the stability of the assay reagents is fundamental to its value in field settings where a cold chain cannot easily be established. This comment allowed us to find a typo in the original submission. Specifically, the lyophilized sensors (split luciferase), controls, and diluent were all stored at room temperature for more than one month before usage in both studies. We have adjusted our methods section to correct the error. The furimazine substrate on the other hand, is more sensitive to storage condition. In liquid form it must be stored at -20C to avoid complete loss of function. In our hands, lyophilized substrate can maintain function for up to 11 days at 4C with a signal loss of 20% (measurements are not included in the manuscript). In our Bangladesh study, we maintained a cold chain for the pilot study to avoid loss of function in the event the shipments were delayed and/or encountered high temperatures along the way.

Of high relevance and obviating our own internal substrate stability measurements, Promega published a study (Hall, MP et al, Anal Chem, 2021, PMID: 33730483) during the development period of our own work, reporting on a new lyophilized formulation with room temperature stability up to one year. Although we were unable to adapt our assay in time, future work can take advantage of improved reagents with high room temperature stability, alleviating these storage limitations. To address this concern, we have updated the manuscript to include the new reference and point out that future studies can proceed without need of cold chain using the reformulated reagents. We thank the reviewer for bringing this to our attention.

3) Can any part of the device/manifold be 3D printed? If so, making the programme/script for 3D printing available to the community would be helpful.

Absolutely. In the original submission we included information about the 3D printer type used for fabrication of the parts (Materials and Methods). We also provided a link to the online Computer-Assisted Design (CAD) documents, from which any reader can access the part files and download them for 3D printing. To further increase accessibility, we have now added a .zip file containing the .stl files which are universally accepted by nearly all 3D printing software.

Sincerely,

Paul Lebel

Staff R&D Engineer, CZ Biohub SF
